# Penile microvascular endothelial function in hypertensive patients: effects of acute type 5 phosphodiesterase inhibition

**DOI:** 10.1590/1414-431X20176601

**Published:** 2018-01-11

**Authors:** V. Verri, A.A. Brandão, E. Tibirica

**Affiliations:** 1 Instituto Nacional de Cardiologia, Ministério da Saúde, Rio de Janeiro, Brasil; 2 Laboratório de Investigação Cardiovascular, Instituto Oswaldo Cruz, Rio de Janeiro, Brasil; 3 Universidade do Estado do Rio de Janeiro, Rio de Janeiro, Brasil

**Keywords:** Microvascular dysfunction, Hypertension, Laser speckle contrast imaging, Penile microcirculation

## Abstract

The primary aim of this study was to evaluate penile endothelial microvascular function in patients with primary arterial hypertension and age-matched normotensive subjects using laser speckle contrast imaging (LSCI). Additionally, we analyzed the acute penile microvascular effects induced by oral phosphodiesterase type 5 inhibitor (sildenafil; SIL) administration. Endothelium-dependent microvascular reactivity was evaluated in the penises and forearms of hypertensive patients (aged 58.8±6.6 years, n=34) and age-matched healthy volunteers (n=33) at rest and 60 min following oral SIL (100 mg) administration. LSCI was coupled with cutaneous acetylcholine (ACh) iontophoresis using increasing anodal currents. Basal penile cutaneous vascular conductance (CVC) values were not significantly different between control subjects and hypertensive individuals. Penile CVC values increased significantly after SIL administration in control (P*<*0.0001) and hypertensive (P*<*0.0001) subjects. Peak CVC values were not different between the two groups during penile ACh iontophoresis before SIL administration (P=0.2052). Peak CVC values were higher in control subjects than in hypertensive subjects after SIL administration (P=0.0427). Penile endothelium-dependent microvascular function is, to some extent, preserved in patients presenting with primary arterial hypertension under effective anti-hypertensive treatment. LSCI may be a valuable non-invasive tool for the evaluation of penile vascular responses to phosphodiesterase type 5 inhibitor.

## Introduction

Arterial hypertension is the result of complex interactions between environmental and genetic factors that initiate and perpetuate blood pressure elevations and is one of the most prevalent cardiovascular diseases worldwide, affecting approximately 25% of the global population. Arterial hypertension is characterized by significant systemic microvascular reactivity alterations, as demonstrated by studies reporting abnormal microvascular endothelial responses to a variety of stimuli in hypertensive patients, responses identified mostly via laser perfusion monitoring technology (1,2). Moreover, several investigators have demonstrated reductions in capillary density and/or reactivity in experimental models of hypertension (3), patients with hypertension (4,5), people with borderline hypertension (6), and normotensive subjects with a familial predisposition for developing hypertension (7). Given that microvascular dysfunction and rarefaction are unquestionably involved in arterial hypertension pathophysiology and that hypertension can lead to microvascular alterations, a bi-directional causality most likely exists between the two conditions.

In addition to having higher cardiovascular risks, hypertensive patients frequently have impaired quality of life because of vasculogenic erectile dysfunction (ED) (8). The prevalence of ED in hypertensive patients is significantly higher than that in age-matched normotensive subjects (8). It is now accepted that vasculogenic ED is associated with systemic vascular endothelial dysfunction and may be considered an early and independent marker of increased cardiovascular risk (9). Moreover, ED is associated with classical atherothrombotic disorder risk factors, such as age, sedentary lifestyle, hypertension, dysglycemia, dyslipidemia, obesity and smoking (10). It has also been suggested that patients with hypertension and ED who do not respond to type 5 phosphodiesterase enzyme (PDE-5) inhibitors may be at increased risk for cardiovascular events (11).

The pathophysiology of ED in hypertensive patients is characterized by vascular endothelial dysfunction caused by reduced nitric oxide (NO) bioavailability and increased oxidative stress resulting from increases in NADPH oxidase activity driven by angiotensin II that ultimately result in reduced corpus cavernosum blood vessel vasodilatory capacity (12). PDE-5 inhibitors have been shown to be effective and safe in patients with cardiovascular disease and to restore penile vasculature vasodilation (13). In patients with arterial hypertension, sildenafil improves ED without exerting any serious adverse effects on blood pressure (13). PDE-5 inhibitors reduce cyclic guanosine monophosphate (cGMP) catabolism, thereby increasing cGMP levels and enhancing vasodilation (14). Moreover, PDE-5 inhibitors significantly increase NO levels (15).

Laser speckle contrast imaging (LSCI) provides an innovative approach for non-invasive evaluation of skin microvascular endothelial function (16). A major advantage of this technique is that LSCI is more reproducible than earlier procedures, such as laser Doppler flowmetry and laser Doppler imaging (17). LSCI has also previously been shown to be an effective noninvasive technique for evaluating systemic microvascular reactivity in patients presenting with cardiovascular and metabolic diseases (16,18). Moreover, we recently showed that LSCI is a valuable non-invasive method for evaluating penile microvascular endothelial function in healthy volunteers (19).

The primary objective of this study was to evaluate penile endothelial microvascular function in hypertensive patients and age-matched normotensive subject using LSCI. The secondary objective of this study was to determine the acute effects of oral sildenafil (100 mg) administration on penile and systemic microvascular endothelial function.

## Material and Methods

### Study design

The present study was undertaken in accordance with the Helsinki Declaration of 1975, revised in 2000, and was approved by the Institutional Review Board (IRB) of the National Institute of Cardiology, Rio de Janeiro, RJ, Brazil, under protocol #CAAE17663813.4.0000.5272 and registered at ClinicalTrials.gov (NCT02620995). Once deemed eligible to participate in this study, all subjects read and signed an informed consent document approved by the IRB.

Thirty-four hypertensive male outpatients who were eligible for this study were recruited from our hypertension clinics and from referrals by colleagues. The clinical characteristics of these patients are presented in Tables 1 and 2. All subjects underwent complete medical examinations, which included searches for target organ lesions. Ambulatory hypertension was diagnosed using a clinically validated Omron Intellisense M7 upper arm blood pressure monitor (Omron Healthcare Europe B.V., Hoofddorp, The Netherlands) as follows: each subject rested for 10 min in the supine position in a cool (23±1°C) and quiet environment before undergoing blood pressure measurements in both arms. The arm exhibiting the higher blood pressure was used for subsequent evaluations. Each patient then underwent measurements in the upright (orthostatic) position and three measurements in the sitting position at 1-min intervals. The mean values of the two last measurements were used in this study. Insulin resistance was measured using the homeostatic model assessment (HOMA-IR) and calculated as the product of fasting plasma insulin (in μUI/mL) multiplied by 0.0555 and as the quotient of fasting plasma glucose (in mg/dL) divided by 22.5.

**Table 1.** Characteristics of healthy volunteers and hypertensive patients.

Parameters	Healthy volunteers (n=33)	Hypertensive patients (n=34)	P value
Age (years)	56.9±5.0	58.8±6.6	0.2102
Body weight (kg)	77.7±11.9	84.6±13.0	**0.0260**
Body mass index (kg/m^2^)	25.9±3.3	29.6±4.4	**0.0004**
Waist circumference (cm)	93.0±8.9	102.0±9.3	**0.0002**
Fasting glucose (mg/dL)	99.4±7.3	102.7±10.2	0.1422
Glycated hemoglobin (%)	5.3±0.4	5.6±0.3	**0.0211**
Fasting insulin (µUI/mL)	7.2 (5.5–10.0)	12.4 (7.4–15.9)	**0.0009**
HOMA-IR index	1.82 (1.36-2.34)	2.99 (1.96-4.25)	**0.0007**
Total cholesterol (mg/dL)	217.4±26.4	201.1±40.3	0.0566
LDL cholesterol (mg/dL)	142.9±25.8	124.0±33.5	**0.0121**
HDL cholesterol (mg/dL)	44.9±8.1	43.3±10.6	0.5140
Triglycerides (mg/dL)	141.0 (95.0-182.0)	155.0 (101.8-212.8)	0.2617
Urea (mg/dL)	31.6±9.4	33.1±9.3	0.4934
Creatinine (mg/dL)	0.9 (0.8-1.0)	0.9 (0.8-1.1)	0.6201
Uric acid (mg/dL)	5.9±1.2	6.5±1.5	0.1001
Microalbumin (mg/L)	5.0 (1.0-7.4)	5.0 (1.5-10.0)	0.4140
hs-CRP	0.14 (0.09-0.21)	0.20 (0.09-0.37)	0.3593
Office SAP (mmHg)	122.6±8.8	136.7±13.8	**<0.0001**
Office DAP (mmHg)	79.3±6.6	86.7±7.6	**<0.0001**
Office MAP (mmHg)	93.7±6.8	103.4±8.7	**<0.0001**
ABPM			
Mean 24-h SAP (mmHg)	–	127.0 (124.0-135.3)	
Mean 24-h DAP (mmHg)	–	82.5 (75.7-86.5)	
Mean daytime SAP (mmHg)	–	130.5 (126.0-135.8)	
Mean daytime DAP (mmHg)	–	85.0 (77.7-88.0)	
Mean nighttime SAP (mmHg)	–	116.0 (112.0-128.3)	
Mean nighttime DAP (mmHg)	–	73.2±9.7	
MAP-F			
PRE (mmHg)	96.3±9.9	108.0±8.6	**<0.0001**
POST (mmHg)	90.3±8.4*	99.3±9.1*	**<0.0001**
Δ MAP-F	-6.0±7.2	-8.8±6.1	0.0980

Results are reported as means±SD. Values that did not follow a Gaussian distribution are reported as medians (25th–75th percentile) (Shapiro-Wilk normality test). P values were estimated using two-tailed unpaired Student's *t*-tests or Mann-Whitney U tests, as appropriate. P values in bold type denote statistically significant differences. MAP values were obtained during a microcirculatory flowmetry (MAP-F) before (PRE) and after (POST) oral administration of a single dose of sildenafil (100 mg). Δ MAP-F indicates the reduction in MAP after sildenafil. ABPM: ambulatory blood pressure monitoring; DAP: diastolic arterial pressure; HOMA-IR: homeostatic model assessment of insulin resistance; hs-CRP: high-sensitivity C-reactive protein; MAP, mean arterial pressure; SAP: systolic arterial pressure.

* P<0.0001, compared with MAP-F PRE.

**Table 2.** Antihypertensive and cardiovascular drugs used by hypertensive patients.

Drugs	Hypertensive patients (n=34)
Angiotensin II receptor blockers	26 (76.5)
Angiotensin converting enzyme inhibitors	9 (26.5)
Calcium channel blockers	17 (50)
β-adrenergic blockers	5 (14.7)
Diuretics	24 (70.6)
Statins	9 (26.5)
Fibrates	2 (5.9)

Data are reported as n (%).

Sexually active male hypertensive patients aged 50 to 70 years with blood pressure levels <160/100 mmHg while receiving anti-hypertensive pharmacological treatment were included in this study. All patients with hypertension were evaluated via 24-h ambulatory blood pressure monitoring using oscillometric devices (Spacelabs, USA). The monitor was installed on the non-dominant arm between 7:00 and 9:00 am and removed 24 h later. Recordings were made every 15 min from 7:00 am to 11:00 pm (diurnal BP values) and every 30 min from 11:00 pm to 7:00 am (nocturnal BP values).

Patients with a history of diabetes mellitus, coronary artery disease, secondary arterial hypertension, neurologic or psychiatric disorders, or decompensated kidney or liver disease were excluded from this study. Patients using nitrates or alpha-adrenergic blockers were also excluded from this study. Patients should not have taken PDE-5 inhibitors within 30 days before enrolling in the study.

Thirty-three age-matched normotensive volunteers who were free of cardiovascular disease served as normotensive controls (Table 1). This group was considered a “reference” group for microvascular parameters. All patients and control subjects underwent detailed histories and physical examinations.

All evaluations were performed in the morning between 8:00 am and 12:00 pm after a 12-h fast. Blood specimens were collected, and subjects were asked to lie down for 20 min in a quiet environment with a constant temperature of 23±1°C. All examinations followed the same sequence, beginning with LSCI microvascular reactivity measurements in the skin of the forearm, followed by the same measurements in the skin of the penis. The microvascular tests were performed before and 60 min after oral sildenafil (100 mg) administration.

### Evaluation of microcirculatory reactivity

The microcirculatory tests were performed in an undisturbed quiet room with a defined stable temperature (23±1°C) after a 20-min rest period in the supine position. The room temperature was monitored and adjusted if necessary using air conditioning, as the outside temperature was usually >25°C. Iontophoresis was performed in the forearm 60 min after oral sildenafil administration and in the penis approximately 15 min after the forearm procedure because it was technically impossible to perform both procedures simultaneously.

### Evaluation of skin microvascular flow and reactivity

Microvascular reactivity was evaluated using an LSCI system with a laser wavelength of 785 nm (PeriCam PSI system, Perimed, Sweden), which enabled us to perform noninvasive and continuous measurements of cutaneous microvascular perfusion changes, measured in arbitrary perfusion units (APU). The images were analyzed using the indicated software (PIMSoft, Perimed).

High-frame-rate LSCI is a recently marketed non-contact technique based on the analysis of the variations in speckle contrast, which allows measurements of fast changes in skin microvascular blood flow over wide skin areas with very good inter-day reproducibility (17). In fact, LSCI measures skin perfusion over wide areas (up to 100 cm^2^) with a high frequency (up to 100 images/s). LSCI thus presents the advantages of good temporal and spatial resolutions (20).

One skin site on the ventral surface of the forearm was randomly chosen for the recordings. Hair, broken skin, areas of skin pigmentation and visible veins were avoided, and two drug-delivery electrodes were installed using adhesive discs (LI 611, Perimed). The following two measurement areas were identified: a measurement area within the electrode (ACh) and another measurement area (baseline control) adjacent to the electrode. A vacuum cushion (AB Germa, Sweden) was used to minimize recording artifacts generated by arm movements. ACh 2% w/v (Sigma Chemical Co., USA) iontophoresis was performed using a micropharmacology system (PF 751 PeriIont USB Power Supply, Perimed, Sweden) with increasing anodal currents of 30, 60, 90, 120, 150, and 180 μA, which were administered for 10-s intervals spaced 1 min apart. The total charges for the above currents were 0.3, 0.6, 0.9, 1.2, 1.5, and 1.8 mC, respectively. The dispersive electrode was attached approximately 15 cm from the electrophoresis chamber. The pharmacological test results are reported as peak values representing the maximal vasodilation observed after the highest Ach dose. Skin blood flow measurements in APU were divided by mean arterial pressure values to yield cutaneous vascular conductance (CVC) in APU/mmHg.

Finally, to evaluate penile skin microcirculation, the electrode was positioned on the base of the penis to perform iontophoretic ACh delivery, as described above and in a previous work (19). In this case, the dispersive electrode was attached to the thigh of each research subject. We used a fenestrated drape with a round opening in the center to lessen the effects of movement on the penile microvascular blood flow recordings. The penis was introduced through the opening of the drape and fixed to the drape using medical pressure-sensitive adhesive tape (Henkel Adhesive North America, USA). This tape features a hypoallergenic adhesive that is designed to hold firmly to the skin and dressing materials but can be easily removed without damaging the skin. The recordings of penile microvascular flow were always performed on a flaccid penis.

### Statistical analysis

The results are reported as means±SD. Values that did not follow a Gaussian distribution are presented as medians (25th-75th percentile; Shapiro-Wilk normality test). The results were analyzed using two-tailed paired or unpaired Student’s *t*-tests, Wilcoxon matched pairs tests, the Mann-Whitney U-test or repeated measures ANOVA followed by Dunnett’s multiple comparison test when appropriate. P values <0.05 were considered to be statistically significant. All statistical analyses were performed using Prism, version 6.0 (GraphPad Software Inc. USA).

## Results

### Clinical characteristics of the study subjects

The clinical characteristics of the healthy volunteers and hypertensive patients, including information regarding current cardiovascular drug use, are presented in Tables 1 and 2. Anthropometric parameters (body weight, body mass index and waist circumference) were significantly higher in hypertensive patients than in normotensive controls.

Systolic, diastolic and mean arterial blood pressure values were significantly higher in hypertensive patients than in normotensive controls. Moreover, mean arterial pressure measured during microvascular flowmetry (MAP-F) was higher in hypertensive patients than in normotensive controls, both before and after sildenafil administration. We also observed a significant decrease in mean arterial pressure after sildenafil administration in both study groups. However, there was no significant difference in the size of this decrease between the two groups (Table 1).

LDL-cholesterol plasma levels were lower in hypertensive patients than in normotensive controls, likely because 26.5% of patients were taking lipid-lowering drugs (statins). No other clinical parameter was different between patients and controls.

### Evaluation of skin microvascular flow and reactivity

**Figure 1.**
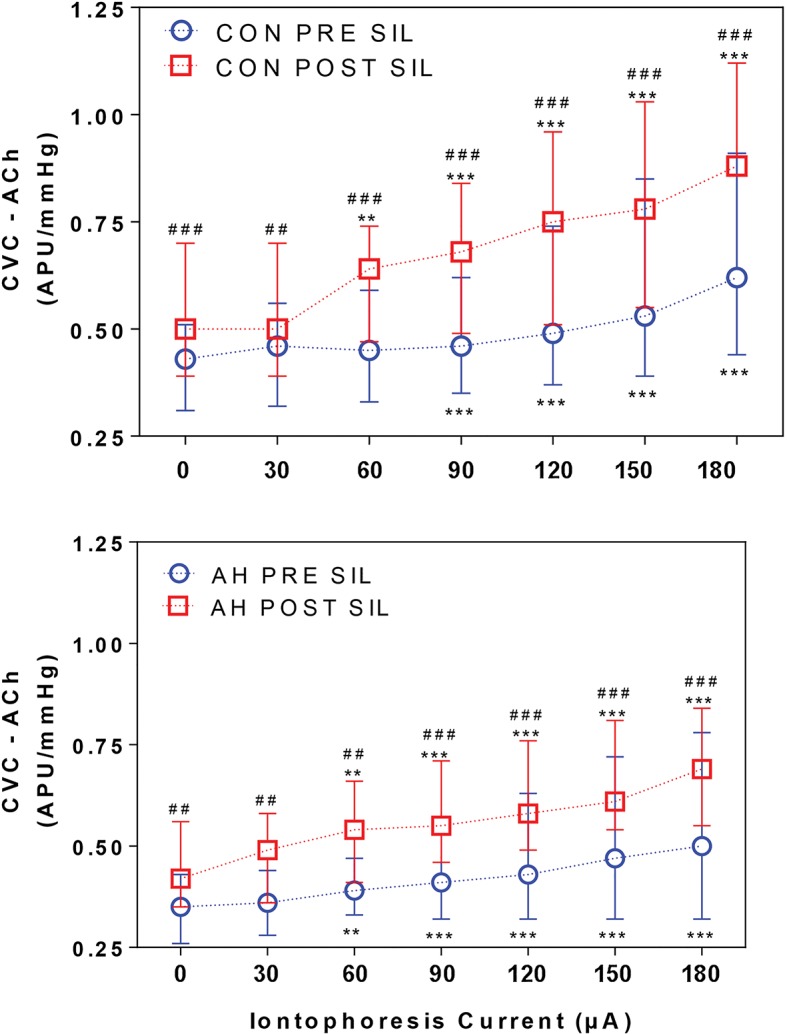
Effects of penile skin acetylcholine (ACh) iontophoresis on cutaneous microvascular conductance (CVC), in arbitrary perfusion units (APU) divided by mean arterial pressure in mmHg in healthy volunteers (upper panel, CON; n=33) and hypertensive patients (lower panel, AH; n=34) before (PRE SIL) and after (POST SIL) oral sildenafil (100 mg) administration. Data are reported as medians (interquartile range) and were analyzed using Wilcoxon’s test, the Mann-Whitney U-test or repeated measures analysis of variance followed by Dunnett’s multiple comparison test when appropriate. **P<0.01 and ***P<0.001 compared with baseline values. ^##^P<0.01 and ^###^P<0.001 compared with PRE SIL.

Penile skin ACh iontophoresis induced significant, current-related increases in microvascular CVC (before and after SIL administration), and CVC was significantly higher after SIL administration than before SIL administration in both healthy controls and hypertensive subjects (Figure 1).

Before SIL administration, the basal penile skin microvascular flow values were 37.0 (28.5–48.5) and 38.0 (27.0–46.2) APU in control and hypertensive participants, respectively (P*=*0.5808). After SIL administration, the flow values were 46.0 (39.0–57.5) and 42.5 (31.5–54.7) APU in control and hypertensive participants, respectively (P*=*0.4256). Penile baseline microvascular flow was significantly increased after SIL administration compared with before SIL administration in control (P*=*0.0006) and hypertensive (P*=*0.0038) subjects. Before SIL administration, the basal penile CVC values were 0.43 (0.31–0.51) and 0.35 (0.26–0.43) APU/mmHg in control and hypertensive participants, respectively (P*=*0.0590). After SIL administration, the flow values were 0.50 (0.39–0.70) and 0.42 (0.35–0.56) APU/mmHg in control and hypertensive participants, respectively (P*=*0.0857). Penile baseline CVC values increased significantly after SIL administration compared with before SIL administration (Figure 1) in control (P*<*0.0001) and hypertensive (P*<*0.0001) subjects.

Before SIL administration, the peak CVC values during penile ACh iontophoresis were 0.62 (0.44–0.91) and 0.50 (0.33–0.79) APU/mmHg in control and hypertensive participants, respectively (P=0.2052). After SIL administration, the peak CVC values during penile ACh iontophoresis were 0.88 (0.66–1.12) and 0.69 (0.56–0.84) APU/mmHg in control and hypertensive participants, respectively (P=0.0427). In control subjects, the peak penile CVC values resulting from ACh iontophoresis were significantly higher after SIL administration than before SIL administration (P<0.0001); the same was true in hypertensive subjects (P=0.0006; Figure 1).

There were no significant correlations between mean arterial pressure changes following sildenafil administration and penile microvascular reactivity (Table 3).

**Table 3.** Correlations between mean arterial pressure changes induced by sildenafil, and endothelium-dependent systemic and penile microvascular reactivity in hypertensive patients.

Parameter	Δ MAP (mmHg)/*r* (P value)
CVC max (APU/mmHg)	
Systemic	-0.268 (0.125)
Penile	-0.268 (0.125)
Δ CVC (APU/mmHg)	
Systemic	-0.254 (0.1480)
Penile	-0.199 (0.2580)

*r*: Spearman's rank correlation coefficient; CVC max: maximum effects of skin acetylcholine iontophoresis on cutaneous microvascular conductance (CVC) in arbitrary perfusion units of flow (APU) divided by mean arterial pressure in mmHg;

Δ CVC: increases in CVC induced by skin acetylcholine iontophoresis compared to baseline values;

Δ MAP: decreases in mean arterial pressure induced by sildenafil.

A subgroup analyses showed that maximum endothelium-dependent penile vasodilation was reduced in the group of patients treated with β-blockers (n=5), compared with the whole group of patients. On the other hand, penile vasodilation in the subgroup of patients treated with statins was comparable to that observed in the whole group of patients (Table 4).

**Table 4.** Effects of penile acetylcholine iontophoresis on endothelium-dependent cutaneous microvascular conductance in hypertensive patients before (PRE SIL) and after (POST SIL) oral sildenafil (100 mg) administration, according to pharmacological treatments.

	CVC max (APU/mmHg)		
	All patients (n=34)	Beta-blockers (n=5)	Statins (n=9)
PRE SIL	0.50 (0.32-0.79)	0.32 (0.26-0.46)#	0.58 (0.42-0.68)
POST SIL	0.69 (0.56-0.84)***	0.44 (0.31-0.54)##	0.57 (0.43-0.72)

Data are reported as medians (interquartile range). CVC max: maximum effects of skin acetylcholine iontophoresis on cutaneous microvascular conductance in arbitrary perfusion units of flow (APU) divided by mean arterial pressure in mmHg.

*** P<0.001 compared with PRE SIL.

# P<0.05 and

## P<0.01 compared with all patients (Mann-Whitney U-test).

### Systemic microvascular reactivity

**Figure 2.**
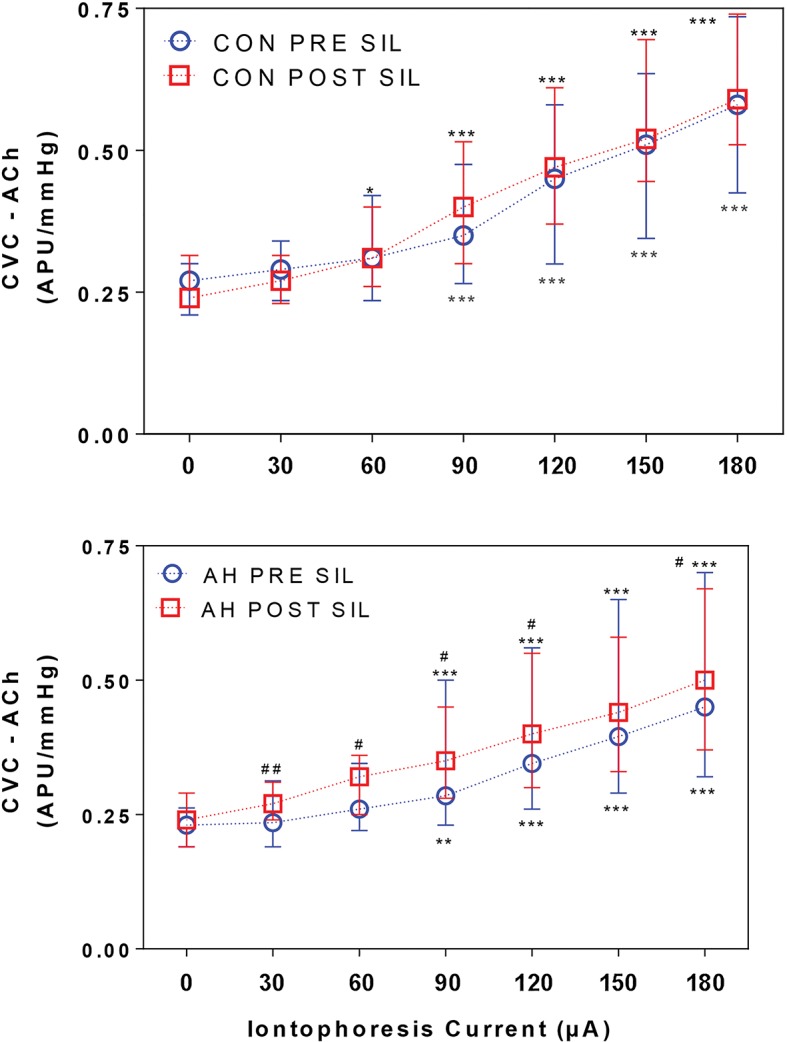
Effects of forearm skin acetylcholine (ACh) iontophoresis on cutaneous microvascular conductance (CVC), in arbitrary perfusion units (APU) divided by mean arterial pressure in mmHg in healthy volunteers (upper panel, CON; n=33) and hypertensive patients (lower panel, AH; n=34) before (PRE SIL) and after (POST SIL) oral sildenafil (100 mg) administration. Data are reported as medians (interquartile range) and were analyzed using Wilcoxon’s test, the Mann-Whitney U-test or repeated measures analysis of variance followed by Dunnett’s multiple comparison test when appropriate. *P<0.05, **P<0.01 and ***P<0.001 compared with baseline values. ^#^P<0.05 and ^##^P<0.01 compared with PRE SIL.

Forearm skin ACh iontophoresis also induced significant, current-related increases in microvascular CVC (before and after SIL administration); however, CVC was higher only in hypertensive subjects following SIL administration (Figure 2).

Before SIL administration, the basal forearm skin microvascular flow values were 25.0 (22.0-28.5) and 25.5 (21.5-28.0) APU in control and hypertensive participants, respectively (P*=*0.6556). After SIL, the flow values were 22.0 (20.0-27.0) and 25.0 (18.7-29.0) APU in control and hypertensive participants, respectively (P*=*0.4177). Forearm baseline microvascular flow was not significantly increased after SIL administration compared with before SIL administration in control (P*=*0.2837) or hypertensive (P*=*0.8222) subjects. Before SIL administration, the basal CVC values were 0.27 (0.21-0.30) and 0.23 (0.19-0.0.26) APU/mmHg in control and hypertensive participants, respectively (P*=*0.0274). After SIL administration, the CVC values were 0.24 (0.21-0.30) and 0.24 (0.19-0.29) APU/mmHg in control and hypertensive participants, respectively (P*=*1.0). Forearm baseline CVC did not change significantly after SIL administration compared with before SIL administration in control (P=0.5494) or hypertensive (P=0.0671) subjects (Figure 2).

Before SIL administration, the peak CVC values during forearm ACh iontophoresis were 0.58 (0.42-0.73) and 0.45 (0.32-0.70) APU/mmHg in control and hypertensive participants, respectively (P=0.1083). After SIL administration, the peak CVC values during forearm ACh iontophoresis were 0.59 (0.51-0.74) and 0.50 (0.37-0.67) APU/mmHg in control and hypertensive participants, respectively (P=0.0378). In control subjects, the peak forearm CVC values resulting from ACh iontophoresis after SIL administration were not significantly different from those resulting from ACh iontophoresis before SIL administration (P<0.3545); however, in hypertensive subjects, there was an increase in peak CVC after SIL administration compared with before SIL administration (P=0.0497; Figure 1).

There were no significant correlations between mean arterial pressure changes following SIL administration and systemic (forearm) microvascular reactivity (Table 3).

## Discussion

The main findings of this study are as follows: i) overall endothelial-dependent penile microvascular vasodilation decreased in hypertensive patients compared with normotensive age-matched control subjects; ii) acute SIL administration significantly increased penile microvascular vasodilation in both normotensive and hypertensive individuals; iii) control subjects experienced greater maximum increases in penile microvascular conductance induced by acetylcholine iontophoresis after SIL administration than hypertensive patients, and iv) SIL increased systemic endothelial-dependent microvascular vasodilation in hypertensive patients, but not normotensive subjects.

In our study, hypertensive patients exhibited greater mean body weight, body mass index and waist circumference than age-matched normotensive individuals. These findings support those of previous studies demonstrating the existence of well-known links between different components of metabolic syndrome, including overweight/obesity and increased visceral fat deposition, and arterial hypertension (21). Moreover, maximum penile microvascular vasodilation was greater in normotensive individuals than in hypertensive patients after sildenafil administration. Although hypertensive patients also exhibited significantly increased microvascular responses to sildenafil, the global endothelium-dependent penile microvascular vasodilatory response induced by cutaneous acetylcholine iontophoresis appeared to be greater in normotensive controls than in hypertensive patients, both before and after sildenafil administration.

It is also important to mention that in hypertensive patients, ED may be a complication of antihypertensive treatment (22). Antihypertensive drugs that block the sympathetic nervous system, including central and peripheral sympatholytics and β-blockers, have been frequently linked to ED (23,24). Several studies suggest that using diuretics and β-blockers other than nebivolol (25) commonly results in ED in hypertensive men (26,27), while using antihypertensive agents that block the renin-angiotensin system, such as angiotensin-converting-enzyme inhibitors (ACE-i) and angiotensin II receptor blockers (ARBs), improves endothelial function and, consequently, erectile function in hypertensive patients (22,23). Calcium-channel blockers (CCB) have been reported to have neutral effects on erectile function (28). Most hypertensive patients enrolled in the present study were receiving antihypertensive drugs that theoretically do not interfere with erectile function (CCBs, 50% of patients) or improve erectile function (ARBs or ACE-is, 100% of patients). Only a small proportion of patients (∼15%) were receiving β-blockers. Most patients (∼70%) were being treated with diuretics for medical reasons. In the present study, subgroup analyses showed that maximum endothelium-dependent penile microvascular vasodilation was significantly reduced compared to that observed in the whole group of patients. In contrast, in the subgroup of patients treated with statins microvascular vasodilation was comparable to that observed in the whole group of patients. Nevertheless, it is important to note that there were only 5 patients treated with β-blockers and nine patients treated with statins in our study; thus, the small sample size, and consequently reduced statistical power, could have biased statistical analysis of subgroups of patients.

Hypertensive patients included in the present study were under chronic and effective anti-hypertensive treatment, thus explaining the significant increase in baseline penile microvascular flow, as well as the substantial vasodilator response of the penile microcirculation to the stimulation with endothelial-dependent vasodilators. In fact, it is well documented that anti-hypertensive drugs that block the renin-angiotensin system induce significant improvements in macro- and microvascular endothelial function (29). In conclusion, the present study shows that penile microvascular reactivity is satisfactory in treated hypertensive patients.

Sildenafil-induced inhibition of PDE-5, an enzyme considered very specific for the vascular smooth muscle of the penis (10), is known to increase NO bioavailability in the penis and its supplying vasculature, resulting in vasodilation and increased blood flow (13). On the other hand, long-term treatment of patients with arterial hypertension with SIL (50 mg 3 times daily) induces significant reductions in systolic and diastolic ambulatory blood pressure (30). In patients with congestive heart failure, SIL treatment improves oxygen uptake, pulmonary vascular resistance, cardiac output, and exercise capacity (31). Consequently, PDE-5 inhibitors have been proposed for the treatment of several cardiovascular diseases (15,31).

In contrast to the penile microcirculation, systemic microvascular endothelium-dependent vasodilation was not affected by acute SIL administration in normotensive subjects. However, in hypertensive patients, acute SIL administration induced significant increases in systemic microvascular vasodilation, although reduction in arterial pressure was not correlated to microvascular vasodilation increases. These results may be explained by the fact that NO bioavailability is significantly reduced in arterial hypertension, whereas oxidative stress is increased, leading to macrovascular and predominantly microvascular endothelial dysfunction, which is a hallmark of primary arterial hypertension pathophysiology (32,33). Thus, acute increases in systemic NO bioavailability induced by SIL may enhance NO-mediated microvascular vasodilation in hypertensive patients, but not normotensive individuals. We and other groups have previously reported on functional and structural microvascular rarefaction, as well as reduced capillary recruitment, in spontaneously hypertensive rats (34,35) and hypertensive patients (1,5,36). Thus, it is reasonable to assume that although mean arterial blood pressure decreased in both experimental groups, and the reductions were not different between the groups, acute SIL administration improved systemic microvascular function in hypertensive patients, but not in normotensive individuals, via endothelium-dependent recruitment of previously closed microvessels. These findings suggest that systemic microvasculature responds differently to acute SIL administration than penile vasculature.

LSCI may well be a valuable non-invasive tool for the evaluation of penile microvascular responses to PDE-5 inhibitors in patients presenting with cardiovascular diseases.
